# Discovering the inner voice: self-awareness among secondary school learners in Limpopo province

**DOI:** 10.3389/fpsyg.2026.1796963

**Published:** 2026-06-10

**Authors:** T. C. Masutha, M. Mabaso, R. Ramalisa, K. J. Shirindza

**Affiliations:** 1 Department of Advanced Nursing Science, University of Venda, Faculty of Health Sciences, Thohoyandou, Limpopo Province, South Africa; 2 Department of Nursing, Faculty of Health Sciences, University of Pretoria, Pretoria, Gauteng Province, South Africa

**Keywords:** adolescents, inner voice, personal growth, secondary school learners, self-awareness

## Abstract

**Introduction:**

Adolescent mental health is a worldwide public health issue that encompasses the capacity to manage day-to-day challenges. A person’s self-esteem, or how they view themselves, affects their mental health. Self-awareness is a crucial skill that fosters empathy, responsible decision-making, and emotional resilience within the broader Social and Emotional Learning framework. The study aimed to explore how secondary school learners in Limpopo perceive and communicate self-awareness.

**Methods:**

This study employed qualitative participatory action research with an exploratory, descriptive design to examine how learners understood self-awareness, self-esteem, and self-esteem-boosting techniques. Rather than measuring variables, qualitative research focuses on the in-depth exploration of participants’ experiences and viewpoints. The study was conducted at Eiland Forever Resort in the Tzaneen area of the Mopani District, Limpopo Province, during the Ubuntu Community Model of Nursing boot camp, hosted by the University of Pretoria in collaboration with the University of Venda and the University of Limpopo for the six selected secondary schools in Vhembe and Mopani Districts. Forty-eight learners were purposively sampled and participated in the study, comprising 24 males and 24 females from grades nine through 11. A reflective writing exercise and group discussion with three structured questions were used to gather data, and the information was recorded with the participants’ permission. Braun and Clarke’s six-step framework was employed for the thematic analysis, enabling the identification of patterns and the creation of themes and sub-themes. Criteria for trustworthiness and ethical aspects were adhered to throughout the study.

**Results:**

Five themes with their sub-themes emerged: exploring learners’ understanding of self-awareness; learners’ weaknesses; learners’ strengths; describing learners’ future and career-oriented goals; and methods for addressing learners’ weaknesses. Each theme was further supported by several sub-themes that provided deeper insights into the learners’ personal and developmental journeys.

**Conclusion:**

According to the study’s findings, learners have a developing yet uneven grasp of self-awareness, which is crucial for determining their academic commitment, personal growth, and future goals. Self-reflection, self-belief, and knowledge of one’s emotions and talents are fundamental to how learners perceive their experiences at school and beyond, according to findings on self-awareness. Nevertheless, there are several weaknesses, such as emotional, social, academic, and contextual issues that prevent full engagement and outstanding performance, frequently undermining this understanding.

## 1 Introduction

Self-awareness is widely recognized as an important component of learners’ academic and personal development. It refers to the ability to recognize and understand one’s emotions, values, strengths, and limitations, and to reflect on how these influence behavior and learning. According to the Collaborative for Academic, Social, and Emotional Learning (2020), self-awareness is one of the core competencies within the Social and Emotional Learning framework and plays an important role in the development of responsible decision making, empathy, and emotional resilience. The Social and Emotional Learning framework includes five interrelated competencies, namely self-awareness, self-management, social awareness, relationship skills, and responsible decision making, which collectively support learners’ holistic development. These competencies do not develop in isolation but rather influence and strengthen one another over time. In this process, self-awareness plays a foundational role, as learners need to understand their own thoughts, emotions, and behaviors before they are able to manage themselves effectively or engage meaningfully with others (Durlak et al., 2011). Barkoukis et al. (2025) further explain that self-awareness influences how learners develop motivation, self-regulation, and adaptive learning practices, which are essential for successful engagement in school.

Within the broader Social and Emotional Learning framework, self-awareness is closely linked to other self-related constructs such as self-concept, self-efficacy, and emotional intelligence. Self-concept refers to how individuals perceive and evaluate themselves, while self-efficacy describes a person’s belief in their ability to successfully perform specific tasks. Emotional intelligence involves the ability to recognize, understand, and regulate one’s own emotions and respond appropriately to the emotions of others. These related concepts contribute to learners’ ability to reflect on their experiences and develop a clearer understanding of themselves (Goleman, 1995, 2018; Brackett et al., 2019). During adolescence, a developmental stage characterized by identity exploration and increasing social and academic demands, the development of self-awareness becomes particularly important. Santrock (2021) notes that adolescents begin to question who they are, what their strengths are, and how they fit into their social and educational environments. Steinberg (2017) further emphasizes that this stage of development is marked by important emotional and cognitive changes that shape how young people understand themselves and respond to the world around them. As learners deepen their reflection on their identities and experiences, they become better positioned to set realistic goals, manage challenges, and participate meaningfully in their learning. Despite the recognized importance of self-awareness, its development is often influenced by contextual and environmental conditions. In South Africa, particularly in the province of Limpopo, many learners experience structural and psychosocial challenges such as socioeconomic disadvantage, limited access to counseling or psychosocial support services, overcrowded classrooms, and pressures from family or social environments. These challenges can limit opportunities for reflection and personal development, making it harder for learners to fully engage in their own growth. The Department of Basic Education (DBE) (2019, 2022) has emphasized the importance of life skills development and learner wellbeing through the Life Orientation curriculum. However, research suggests that the implementation of these programs is often inconsistent, particularly in under-resourced schools where teachers may lack sufficient training or resources to effectively support learners’ social and emotional development (Omidire et al., 2021). At the same time, studies highlight the importance of supportive educational environments in promoting self-awareness and other social and emotional competencies. Positive relationships with teachers, opportunities for open discussion and reflection, and learning environments that encourage participation can strengthen learners’ ability to understand themselves and regulate their emotions. Rubab et al. (2026), for example, found that a supportive school climate plays an important role in shaping learners’ experiences and promoting meaningful engagement with learning. Similarly, Mngomezulu and Mkhize (2022) suggest that environments that encourage dialog, reflection, and learner participation can support the development of self-awareness and self-empowerment.

Recent research in South Africa has also highlighted the role of related psychological constructs in learners’ academic and personal success. Stols et al. (2024) found that emotional intelligence is a strong predictor of academic performance in certain subjects, demonstrating the importance of emotional awareness and regulation for effective learning. In another study, Masinga (2025) reported that self-concept plays an important role in shaping learners’ aspirations and academic achievement. These findings suggest that the way learners see and understand themselves can influence their motivation, engagement in school, and future aspirations. However, there is still limited research on how learners themselves interpret and express their self-awareness, particularly in under-resourced educational contexts.

Understanding how learners reflect on their experiences, recognize their strengths and limitations, and think about their future aspirations can provide valuable insight into their personal and educational development. Exploring learners’ inner voice, understood as the internal dialog through which individuals interpret their experiences, reflect on their identities, and make sense of their goals (Morin, 2011), offers a way of gaining deeper insight into how learners make meaning of their lives within the social and educational contexts in which they live and learn. The purpose of this study was to explore how secondary school learners in Limpopo Province perceive and express self-awareness within their educational and personal contexts. Specifically, the study aimed to explore how learners reflect on their strengths and limitations to develop self-awareness, how this awareness may help them identify areas for personal improvement, and how learners connect their self-understanding with their future aspirations and career goals.

By focusing on learners’ reflections about themselves and their future goals, this study contributes to a deeper understanding of how self-awareness supports adolescent development. Developing self-awareness may enable learners to make informed decisions, set meaningful goals, and navigate academic and social challenges more effectively, ultimately supporting their broader social, emotional, and educational development.

## 2 Materials and methods

### 2.1 Research approach and designs

This study used qualitative participatory action research with an exploratory, descriptive design to explore how learners understand and express self-awareness. Participatory Action Research (PAR) is an approach within social science research that combines research, action, and participation to address real-life problems while involving the people affected by those problems (Chevalier, 2019). 1. Learners and the researcher investigated issues or problems. 2. Researchers and learners were co-creating knowledge. 3. Researchers facilitated discussion as learners carried out the planned activities. 4. Researchers observed participation and behavior during the feedback-giving session. 5. Researchers evaluated the reflections by learners. Qualitative research focuses on exploring participants’ experiences, perspectives, and meanings in depth rather than measuring variables numerically (Creswell and Poth, 2018). This approach was suitable for the study because it allowed the researchers to capture learners’ reflections on their self-understanding, strengths, weaknesses, and aspirations within the context of a self-awareness boot camp. The design enabled insight into how learners interpret their personal experiences and make sense of their own development.

### 2.2 Study setting

The study was conducted at Eiland Forever Resort in the Tzaneen area of the Mopani District in Limpopo Province during a learner development boot camp organized under the Ubuntu Community Model of Nursing. The boot camp was hosted by the University of Pretoria, in collaboration with the University of Venda and the University of Limpopo, as part of a community engagement initiative to support the holistic development of secondary school learners. Learners from six secondary schools in the Vhembe and Mopani Districts attended the boot camp. The program was designed to create opportunities for learners to participate in activities that encourage personal reflection, teamwork, and leadership development. The boot camp included guided discussions, reflective exercises, and interactive activities focused on self-awareness, self-confidence, goal-setting, and personal responsibility. The environment provided learners with a supportive space to reflect on their strengths, challenges, and future aspirations while interacting with peers from different schools.

### 2.3 Sampling and sample size

Sampling was conducted in three phases.

In the first phase, Vhembe and Mopani Districts were purposively selected because they include schools that participate in the Ubuntu Community Model project.

In the second phase, six secondary schools were purposively selected because they have been adopted by the Ubuntu project. For confidentiality reasons, the names of the schools are not disclosed and are instead referred to as School 1, School 2, School 3, School 4, School 5, and School 6. In the third phase, learners were selected using a combination of purposive and convenience sampling. Teachers and School Governing Body members who serve as Ubuntu ambassadors assisted with the recruitment process. Learners were informed about the boot camp, and those who were interested volunteered to participate. A first come first served approach was used until eight learners were selected from each school. A total of 48 learners participated in the study. The participants were 24 male and 24 female learners. The learners were in Grades 9 to 11 and were approximately 14 to 17 years old. Participants came from rural communities in the Vhembe and Mopani Districts, which allowed for a range of perspectives on their experiences and understanding of self-awareness.

### 2.4 Data collection

Data were collected during the boot camp through reflective writing exercises and group discussions. These methods were chosen because they allowed learners to express their thoughts both individually and through interaction with others. Reflective writing provided learners with an opportunity to privately reflect on their personal experiences and perceptions, while group discussions allowed them to share their views and learn from their peers’ perspectives.

The recruitment process was facilitated through the Ubuntu project, which has existing partnerships with the participating schools. Teachers and School Governing Body members who act as Ubuntu ambassadors assisted in informing learners about the boot camp and inviting them to participate voluntarily.

All learners who attended the boot camp participated in the reflective writing exercise. During this activity, learners were asked to reflect on their personal strengths and weaknesses and consider ways to improve themselves. For example, learners were asked to respond to prompts such as describing their strengths and areas they would like to improve, and how these may influence their future goals. They were also asked to identify goals they hoped to achieve in the future and to consider a possible timeframe for achieving them.

Following the written exercise, learners took part in facilitated group discussions. To ensure discussions remained manageable and that all participants had an opportunity to contribute, learners were divided into smaller groups. During these discussions, learners were guided by three questions designed to explore their understanding of self-awareness.

The questions asked were the following:

What is your understanding of self-awareness?

Why is it important to be self-aware?

How can a person develop a more positive understanding of themselves?

With the participants’ permission, the discussions were audio-recorded to ensure their responses were accurately captured. The written worksheets and audio recordings were stored securely for later analysis. The researchers were involved in the Ubuntu Community Model project and engaged learners during the boot camp. This positioning enabled the researchers to create a supportive, familiar environment for participation. At the same time, care was taken to remain reflective and mindful of the researchers’ roles in shaping the research process, particularly in relation to learners’ backgrounds and the context in which the data were collected.

### 2.5 Data analysis

The data included learners’ written reflections and verbatim transcripts of recorded group discussions. These data were analyzed using thematic analysis following the framework developed by Braun and Clarke (2019, 2020). Thematic analysis is commonly used in qualitative research to identify and interpret patterns of meaning within textual data.

The first stage of the analysis involved familiarization with the data. The researchers read and reread the written responses and discussion transcripts to gain a comprehensive understanding of the learners’ reflections.

In the second stage, initial codes were generated by identifying meaningful segments of the data that captured learners’ important ideas.

During the third stage, the researchers examined the codes and began grouping related codes together to form potential themes that represented broader patterns within the data.

The fourth stage involved reviewing and refining the themes to ensure they accurately reflected the data and were distinct from one another.

In the fifth stage, the themes and subthemes were clearly defined and named to capture the key ideas emerging from learners’ reflections.

The final stage involved producing the report by selecting quotations from the participants that clearly illustrated each theme and subtheme. These quotations were used to present learners’ perspectives in their own words and to support the interpretation of the findings.

This systematic process enabled the researchers to move from raw data to meaningful themes that capture learners’ experiences and perspectives on self-awareness.

### 2.6 Ethical considerations

Participation was voluntary, with assent forms signed by parents, as the learners are still minors (ages 14–17). Confidentiality and anonymity were maintained, and written responses were securely stored. Identifiers were removed during transcription to protect participants’ privacy. The Ubuntu project has an ethical clearance number: FHS/21/PH/17/131D. The following criteria for trustworthiness were adhered to: credibility, dependability, confirmability, transferability, and authenticity.

## 3 Findings

### 3.1 Participants’ characteristics

The study explored self-awareness among school learners. A total of 48 participants took part in the study, representing six different schools. From each school, eight learners participated, comprising four females and four males, resulting in a balanced gender distribution of 24 girls and 24 boys. The participants were between 14 and 17 years old and enrolled in Grades 9–11. This age group represents a significant developmental stage characterized by identity exploration, emotional growth, and the emergence of self-awareness. The diversity of participants across schools and grades provided a comprehensive understanding of how learners experience and develop self-awareness within their educational environments.

To ensure confidentiality, participants were assigned codes in the findings section. For example, references such as P1, P2, or S1 indicate participant numbers, while letters such as S1, S2, or similar refer to the school from which the participant was drawn. This coding system protected participants’ identities while allowing the reader to trace the source of each response (Table 1).

**Table 1:** presents a summary of the participants’ characteristics, including gender, grade, school, and district.

Participant code	Gender	Grade	School	District
P1 (S1)	Female	Grade 9	School 1	Vhembe
P2 (S1)	Male	Grade 9	School 1	Vhembe
P3 (S2)	Female	Grade 10	School 2	Mopani
P4 (S2)	Male	Grade 10	School 2	Mopani
P5 (S3)	Female	Grade 11	School 3	Vhembe
P6 (S3)	Male	Grade 11	School 3	Vhembe
P7 (S4)	Female	Grade 9	School 4	Mopani
P8 (S4)	Male	Grade 9	School 4	Mopani

Only a sample of participant codes is shown; all 48 participants were coded similarly.

### 3.2 Presentation of the findings

Five themes with their sub-themes emerged: exploring learners’ understanding of self-awareness; learners’ weaknesses; learners’ strengths; describing learners’ future and career-oriented goals; and methods for addressing learners’ weaknesses. Each theme was further supported by several sub-themes that provided deeper insights into the learners’ personal and developmental journeys.

These sub-themes included self-discovery, self-belief, boosting self-esteem, inability to participate in extramural activities, emotional and academic challenges, physical challenges, and personal, physical, emotional, and academic strengths. Furthermore, learners expressed aspirations under the following sub-themes: career-driven, entrepreneurial, and social welfare goals, and identified personal strategies, support systems, and self-improvement approaches. These themes and sub-themes are discussed in Table 2.

**Table 2:** Themes and sub-themes.

Themes	Sub-themes
Theme 1: Exploring learners’ understanding regarding self-awareness	Self-discovery; Self-belief; Boosting self-esteem
Theme 2: Exploring learners’ weaknesses	Lack of skills to participate in social activities; Emotional factors; Academic challenges; Physical challenges
Theme 3: Exploring learners’ strengths	Personal; Academic; Social strengths
Theme 4: Describing learners’ future and career-oriented goals	Career-driven goals; Entrepreneurial goals; Social welfare goals
Theme 5: Methods of addressing learners’ weaknesses	Personal strategies: Support needed; Emotional management; Social improvement; Self-awareness development

#### 3.2.1 Theme 1: exploring learners’ understanding regarding self-awareness

This refers to examining how students understand, interpret, and comprehend the concept of self-awareness in both academic and personal contexts.

This theme explores how learners understand and interpret self-awareness within both academic and personal contexts. The findings align with social and emotional learning (SEL), particularly the competency of self-awareness, which involves recognizing one’s strengths, limitations, emotions, and values. Three sub-themes emerged, namely self-discovery, self-belief, and self-esteem.

##### 3.2.1.1 Sub-theme 1: self-discovery

A total of 20 participants described self-awareness as the ability to understand their strengths and weaknesses and to use this understanding to guide their decisions. Learners reflected on recognizing their capabilities and limitations, showing an emerging sense of identity and personal insight. This aligns with the SEL construct of self-awareness, particularly the ability to accurately assess one’s strengths and areas for growth. For instance, one participant explained that “self-awareness is the ability to understand yourself, your strengths and weaknesses, and use that knowledge to make better decisions in life and in school” (P13 S2), while another referred to knowing what one is capable of doing, such as learning new skills, as a way to grow both academically and personally (P22 S3). These reflections suggest that self-discovery plays an important role in supporting informed decision-making and ongoing personal development.

*“…Self-awareness is the ability to understand yourself, your strengths and weaknesses, and use that knowledge to make better decisions in life and in school.” …P13 S2.*

*“…Knowing what you are capable of doing, such as speaking English or learning new skills, helps you grow and improve academically and personally.” …P22 S3.*

*“…Staying true to yourself and reflecting on your experiences helps you build confidence and understand who you really are.” …P36 S4.*

The above narratives suggest that self-discovery enables learners to understand themselves in both personal and academic contexts, thereby supporting better decision-making and personal growth.

##### 3.2.1.2 Sub-theme 2: self-belief

A total of 16 participants linked self-awareness to confidence in their abilities and a sense of self-worth. Learners described how believing in themselves influences their willingness to participate, express ideas, and engage with challenges. This reflects the SEL component of self-efficacy, which is closely tied to motivation and persistence. For example, one participant noted that having confidence and good communication skills allows them to express their ideas clearly and believe in themselves when facing challenges (P27 S3), while another explained that seeing oneself as capable increases motivation to work hard and achieve goals (P29 S4). These findings indicate that self-belief strengthens learners’ confidence and motivation, supporting both academic engagement and personal resilience.

*“…Having good communication skills and confidence allows me to express my ideas clearly and believe in myself when I face challenges.” …P27 S3.*

*“…When you see yourself as worthy and capable, you are more motivated to work hard and achieve your goals.” …P29 S4.*

*“…Valuing yourself and believing in your abilities helps you stay focused and overcome difficulties.” …P47 S6.*

The above narratives indicate that self-belief strengthens motivation, self-respect, and confidence, thereby supporting learners’ academic and personal development.

##### 3.2.1.3 Sub-theme 3: self-esteem

A total of 12 participants highlighted self-acceptance and avoiding comparisons with others as key aspects of self-awareness. Learners reflected on how valuing themselves contributes to a positive self-image and emotional well-being, which aligns with the SEL dimension of emotional self-awareness. One participant expressed that knowing and accepting oneself, while avoiding comparison with others, helps to maintain confidence (P1 S1), while another noted that self-acceptance improves confidence and self-esteem (P4 S1). These reflections suggest that self-esteem contributes to emotional stability and supports personal growth by fostering a positive self-concept.

*“…You should know yourself, accept your strengths and weaknesses, and stop comparing yourself with others because it lowers your confidence.” …P1 S1.*

*“…Accepting yourself the way you are and loving yourself helps you feel more confident and improves your self-esteem.” …P4 S1.*

*“…When you stop comparing yourself with others and focus on your own growth, your self-esteem increases.” …P6 S1.*

The above narratives indicate that self-acceptance and self-love are essential in improving self-esteem and developing a positive self-image.

#### 3.2.2 Theme 2: exploring learners’ weaknesses

This theme reflects learners’ awareness of their limitations across academic, emotional, social, and physical contexts. These findings relate to SEL competencies such as self-awareness and self-management, as learners identify challenges that influence their overall functioning and development.

##### 3.2.2.1 Sub-theme 1: lack of skills to participate in social activities

A total of 14 participants reported limited ability to engage in social and extracurricular activities, which in turn affected their confidence and sense of belonging. Learners described difficulties participating in activities such as sports and group interactions, pointing to gaps in relationship skills. For instance, one participant stated that they cannot participate in social activities like sports, which makes them feel less confident around others (P1 S1), while another noted difficulty engaging in activities such as football or swimming (P2 S1). These findings suggest that limited participation reduces opportunities for social interaction and may negatively affect peer integration.

*“…I cannot dance or participate in social activities like sports, and this makes me feel less confident around others.” …P1 S1.*

*“…I cannot play football or swim, which limits my participation in activities with my peers.” …P2 S1.*

*“…I struggle with activities like cooking and debating, so I often avoid group participation.” …P5 S1.*

These narrations indicate that a lack of physical and social skills limits learners’ participation and affects their sense of belonging.

##### 3.2.2.2 Sub-theme 2: emotional factors

A total of 17 participants identified emotional challenges such as shyness, anxiety, and low confidence. Learners reported difficulties expressing themselves and interacting with others, reflecting challenges in emotional regulation within the SEL framework. For example, one participant reported feeling nervous when speaking in front of others and struggling to express themselves clearly (P37 S5), while another indicated difficulty socializing due to shyness (P16 S2). These findings suggest that emotional challenges can hinder learners’ ability to engage effectively in both social and academic settings.

*“…I feel nervous when speaking in front of people and often struggle with expressing myself clearly.” …P37 S5.*

*“…I find it difficult to socialize and interact with others because I am shy and easily hurt.” …P16 S2.*

*“…I am not confident enough to perform or speak without preparation, and this affects my interactions.” …P28 S4.*

The above narratives indicate that emotional factors influence learners’ ability to interact and engage socially and academically.

##### 3.2.2.3 Sub-theme 3: academic challenges

A total of 15 participants reported difficulties related to subject content, concentration, and time management. These challenges reflect limitations in self-management, particularly in areas such as organization and persistence. One participant explained that time management is a challenge and sometimes leads to incomplete work (P26 S4), while another highlighted difficulty concentrating for extended periods (P34 S5). These findings indicate that academic challenges can negatively impact both performance and motivation.

*“…I struggle with Physics and often receive low marks, which affects my confidence in the subject.” …P41 S6.*

*“…Time management is a problem for me, and I sometimes fail to complete my work on time.” …P26 S4.*

*“…I find it difficult to read and concentrate for long periods.” …P34 S5.*

These narratives suggest that academic challenges impact learners’ motivation and academic performance.

##### 3.2.2.4 Sub-theme 4: physical challenges

A total of 9 participants identified physical limitations that affect their participation and productivity. Learners reported issues such as low physical strength and health concerns that affect their daily functioning. For example, one participant indicated that low physical strength makes it difficult to participate in activities (P18 S3), while another noted that low energy levels affect productivity (P39 S5). These findings suggest that physical challenges can influence learners’ engagement and overall participation.

*“…I have low physical strength, which makes it difficult for me to participate in activities.” …P18 S3.*

*“…I experience breathing difficulties that affect my energy levels.” …P22 S3.*

*“…I sleep too much and sometimes feel lazy, which affects my productivity.” …P39 S5.*

The above narrations indicate that physical challenges can limit learners’ engagement and performance.

#### 3.2.3 Theme 3: exploring learners’ strengths

This theme highlights learners’ recognition of their positive qualities and abilities across personal, academic, and social contexts. These strengths align with SEL competencies such as self-awareness, self-management, and relationship skills, and contribute to overall development.

##### 3.2.3.1 Sub-theme 1: personal strength

A total of 17 participants identified personal attributes such as confidence, positivity, and communication skills as key strengths. Learners described their ability to remain positive and express themselves effectively in different situations. For instance, one participant noted having high self-esteem and confidence when facing challenges (P36 S5), while another highlighted confidence in communicating with others (P40 S5). These findings suggest that personal strengths support emotional resilience and enable learners to cope effectively with challenges.

*“…I have high self-esteem and believe in myself when facing challenges.” …P36 S5.*

*“…I am confident and able to communicate well with others.” …P40 S5.*

*“…My ability to stay positive helps me overcome difficult situations.” …P30 S4.*

These narratives suggest that personal strengths help learners manage challenges effectively.

##### 3.2.3.2 Sub-theme 2: academic strength

A total of 15 participants described strengths in understanding concepts, learning quickly, and managing their time effectively. These abilities reflect strong self-management skills. One participant indicated that they can easily grasp new information and learn quickly (P45 S6), while another noted that effective time management helps them complete their schoolwork efficiently (P35 S5). These findings suggest that academic strengths contribute positively to performance and learning outcomes.

*“…I am good at solving chemistry problems and understanding complex concepts.” …P23 S3.*

*“…I can easily grasp new information and learn quickly.” …P45 S6.*

*“…I manage my time well, which helps me complete my schoolwork effectively.” …P35 S5.*

These strengths support academic performance and learning efficiency.

##### 3.2.3.3 Sub-theme 3: social strength

A total of 11 participants highlighted strengths in social interaction and in engaging in activities. Learners described participation in sports and recreational activities as a way to build confidence and strengthen relationships. For example, one participant stated that playing soccer helps them interact with others and build friendships (P11 S2), while another noted that activities such as dancing and swimming improve confidence and connection with others (P9 S2). These findings indicate that social engagement plays an important role in strengthening interpersonal relationships and fostering a sense of belonging.

*“…I enjoy playing soccer, which helps me interact with others and build friendships.” …P11 S2.*

*“…Swimming and dancing help me feel confident and connect with people.” …P9 S2.*

*“…Dancing always cheers me up and helps me interact with others.” …P37 S5.*

The above information suggests that engaging in recreational activities strengthens social ties.

#### 3.2.4 Theme 4: describing the learners’ future and career-oriented goals

This is to investigate and record students’ goals, intentions, and aspirations for their future academic, professional, and personal paths.

This theme explores learners’ aspirations and future, reflecting their motivation, values, and sense of purpose. These findings align with SEL competencies such as self-awareness and responsible decision-making.

##### 3.2.4.1 Sub-theme 1: career-driven goals

A total of 22 participants expressed aspirations to pursue professional careers across various fields, including healthcare, law, and engineering. Learners demonstrated clear planning and a strong focus on long-term goals. For example, one participant described plans to complete matric, pursue a law degree, and achieve success within 10 years (P31 S5), while others expressed ambitions in medical and scientific fields (P47 S6). These findings suggest that many learners are strongly motivated by professional success and long-term personal development.

*“…To become a nuclear medical technologist, and if that doesn’t work, I would become a model or a designer,” …P23 S3.*

*“I want to be a vet, since I am aiming to save animals in my surrounding area, as we know many animals are facing difficulties and end up dying, so I want them to live a long life.” …P47 S6.*

*“…Currently, I am in grade 11. Next year, I will write my matric pass and apply for law. In 5 years’ time, I will be at university studying for my law degree. In 10 years, I will help my community, family, and be successful” … P31 S5.*

The narrations above indicate that most learners expressed ambitions to pursue careers in various professional and scientific fields.

##### 3.2.4.2 Sub-theme 2: entrepreneurial goals

A total of 11 participants expressed aspirations to start and manage their own businesses. These goals reflect a desire for independence, leadership, and financial growth. One participant indicated a desire to become a CEO and run a successful company (P29 S4), while another highlighted the goal of owning a business to achieve financial stability (P27 S3). These findings suggest that some learners are motivated by autonomy and innovation.

*“…I want to become a CEO and run my own company successfully.” …P29 S4.*

*“…I would like to own a business where I can help people and grow financially.” …P27 S3.*

These narratives reflect a desire for independence and leadership.

##### 3.2.4.3 Sub-theme 3: social welfare goals

A total of 13 participants expressed goals focused on helping others and contributing to their communities. Learners described aspirations to support their families and improve the lives of others. For instance, one participant stated a desire to help people in need and support their community (P14 S2), while another emphasized supporting their family (P33 S5). These findings reflect a strong sense of empathy and social responsibility among learners.

*“…I want to help people in need and support my community.” …P14 S2.*

*“…I want to help my family and ensure they have food and support.” …P33 S5.*

*“…I would like to own a farm and provide jobs for unemployed people.” …P27 S4.*

These narrations suggest a strong commitment to social responsibility.

#### 3.2.5 Theme 5: methods of addressing learners’ weaknesses

It refers to strategies, interventions, and support networks intended to help students constructively and developmentally overcome behavioral, emotional, social, or academic obstacles.

This theme focuses on the strategies learners identified to overcome their challenges. These approaches align closely with SEL competencies such as self-management, self-awareness, and responsible decision-making.

##### 3.2.5.1 Sub-theme 1: personal strategy to improve academic weaknesses

A total of 17 participants identified strategies such as improving time management, creating more effective study environments, and engaging in additional learning activities. For example, one participant stated that they plan to improve their time management and avoid wasting time (P35 S5), while another stated that they plan to identify and avoid distractions when studying (P17 S3). These findings suggest that learners are actively taking steps to improve their academic performance.

*“…Figuring out what makes me procrastinate and avoid studying near it, if it is an environment, then find a better place to study” …P17 S3.*

*“…I will improve in time management. I will no longer be a time-waster” …P35 S5.*

*“…I want to improve all my science studies so I can be accepted into every University.” …P22 S3.*

##### 3.2.5.2 Sub-theme 2: support needed

A total of 13 participants emphasized the importance of seeking support from professionals, peers, and other resources. One participant noted that they would seek help from professionals when facing challenges (P6 S1), while another referred to using videos and guidance to improve emotional well-being (P9 S2). These findings highlight the important role that support systems play in learners’ development.

The following quotes are from the participants:

*“…I will seek help from professionals when I face challenges.” …P6 S1.*

*“…Watching helpful videos and asking for guidance can improve my emotional well-being.” …P9 S2.*

*“…I’ll train my body to be flexible and also ask for help from other people” … P12 S2.*

These narratives suggest that support systems play a key role in development.

##### 3.2.5.3 Sub-theme 3: ways to overcome emotional weaknesses

A total of 14 participants identified strategies such as building confidence, improving communication, and managing emotions. For instance, one participant indicated a plan to control their temper and become more patient (P12 S2), while another emphasized surrounding themselves with others to overcome shyness (P19 S3). These findings suggest that learners are developing practical strategies to improve emotional regulation.

*“…I will try to surround myself with people to overcome my shyness and improve my confidence.” …P19 S3.*

*“…I will work on controlling my temper and becoming more patient.” …P12 S2.*

These narrations indicate emotional growth strategies.

##### 3.2.5.4 Sub-theme 4: improving social activities

A total of 11 participants described efforts to improve their social engagement by learning new skills and participating more actively in activities. One participant noted that watching tutorials can help improve confidence and social interaction (P18 S3), while another expressed a desire to practice dancing to improve social skills (P7 S1). These findings indicate that learners are intentionally working to strengthen their social competence. Participants showed willingness to develop social skills.

*“…I will watch tutorials to learn singing and dancing and improve my confidence.” …P18 S3.*

*“…I want to learn how to dance and practice regularly to improve my social skills.” …P7 S1.*

*“…I will start by writing lyrics on a piece of paper and then practice singing” …P6 S1.*

These narratives indicate efforts to improve social engagement.

##### 3.2.5.5 Sub-theme 5: ways to improve self-awareness

A total of 15 participants highlighted strategies such as self-reflection, self-acceptance, and focusing on personal growth. For example, one participant stated that understanding their strengths and weaknesses helps them improve and grow (P41 S6), while another emphasized accepting themselves and focusing on their strengths (P45 S6). These findings suggest that reflective practices play a key role in enhancing self-awareness and supporting personal development.

*“…I will accept myself as I am and focus on my strengths instead of comparing myself to others.” …P45 S6.*

*“…By understanding my strengths and weaknesses, I can improve and grow.” …P41 S6.*

*“…I will motivate myself and continue working on becoming a better version of myself.” …P32 S5.*

These narrations suggest that self-reflection and acceptance are key to improving self-awareness.

## 4 Discussion

The purpose of this study was to explore how secondary school learners in Limpopo Province perceive and express self-awareness within their educational and personal contexts. Specifically, the study aimed to explore how learners reflect on their strengths and weaknesses to develop self-awareness, how this awareness may help them identify areas for personal improvement, and how learners connect their self-understanding with their future aspirations and career goals. *Exploring learners’ understanding of self-awareness,* many learners defined self-awareness as both practical (making decisions based on strengths) and contemplative (thinking about who they are); this is consistent with current research demonstrating that teenage self-awareness and self-esteem are dynamic and responsive to school circumstances and interventions. Better mental health and academic outcomes are predicted by early and ongoing school assistance for self-esteem, particularly for girls who frequently report lower self-esteem in early adolescence (Carlén et al., 2023).

Learners reflected on their strengths and claimed that structured programs (such as self-esteem development, mindfulness, and resilience training) increased their self-confidence. Self-esteem, resilience, and coping have all significantly increased as a result of randomized and pilot interventions, such as online mindfulness and school-based resilience training (Kobkurkul et al., 2024). This suggests that extracurricular or curricular programs can significantly strengthen the “self-belief” component of self-awareness.

*Regarding exploring learners’ weaknesses with inability to participate in social activities*, learners who have social anxiety or low self-esteem frequently avoid class discussions, clubs, and extracurricular activities; peers and professors often mistake this behavior for indifference. International research on adolescents (Kara et al., 2025) reveals that social–emotional development and future educational outcomes are adversely affected by exclusion from activities, and that barriers to school participation are often multifactorial (mental health issues, attention difficulties, and environmental/sensory barriers).

On emotional factors, poor participation and academic declines were often attributed to emotional management issues (anxiety, low mood, impulsiveness). Targeted programs (CBT-informed skills training, emotion-regulation curriculum) reduce emotional symptoms and enhance classroom functioning, according to recent systematic reviews of emotional-regulation therapies, underscoring emotional regulation as a crucial intervention target (Lancastle et al., 2024).

Regarding the academic challenges, learners reported low motivation, poor study habits, and foundational deficits in reading and numeracy, which were often exacerbated by absenteeism and a lack of home support. Research on educational resilience and focused tutoring demonstrates that scaffolded teaching techniques, individualized tutoring and mentoring, and social support enhance academic performance for students who struggle academically; whole-school approaches also lessen the negative effects of disengagement (Llerena Aguilar et al., 2023).

Some students’ engagement and focus were limited by physical health issues or disabilities (fatigue, chronic sickness, sensory/physical impairments). Contemporary literature highlights that when schools adapt the physical or sensory environment and provide reasonable supports, learners with disabilities show improved engagement, an important reminder that “weaknesses” are often interactional (learner-environment) rather than solely individual deficits (Kara et al., 2025).

Resilience, problem-solving, perseverance, and moral/ethical grounding were identified by learners as personal strengths. According to recent research (Carlén et al., 2023), linking resilience and self-esteem, boosting self-esteem early on helps sustain positive trajectories of wellbeing and adaptive functioning, supporting the notion that personal strengths can be purposefully fostered.

*During the exploration of learners’ strengths,* several students identified academic strengths such as intrinsic interest in subjects, efficient study techniques, and subject-specific competencies (e.g., mathematics or languages). According to the literature (Llerena Aguilar et al., 2023), motivation and broader academic self-efficacy increase when these subject strengths are recognized and developed (through mastery experiences and tailored instruction).

Collaboration abilities, peer leadership, and social skills were identified as critical assets. Strengthening social–emotional skills strengthens relationships, lowers risk behaviors, and increases classroom involvement, according to evidence from school-based interventions. Whole-school programs that intentionally cultivate social skills produce benefits across behavior and academic domains (Lekamge et al., 2025).

*Describing learners’ future and career-oriented goals,* the future-focused narratives of learners were divided into three categories: (a) traditional career goals (professional job, university admission), (b) entrepreneurial goals (start-ups, small enterprises), and (c) social-welfare goals (work that supports disadvantaged groups or the community). Goal feasibility and orientation were influenced by contextual factors, including home resources, local labor markets, and pandemic-related interruptions. Studies conducted in South Africa during and after the COVID-19 pandemic reveal changes in goals and a rise in interest in vocational and entrepreneurial pathways where formal employment appears uncertain. However, they also point out inadequacies in access to entrepreneurship training and career counseling (Mangwedi, 2022).

*Regarding methods of addressing learners’ weaknesses*, learners indicated that when paired with mentorship, practical skills training, and connections to local opportunities, career counseling and entrepreneurship instruction in schools, learners turn their aspirations into actionable plans. According to studies, incorporating entrepreneurial skills and career discussions into the curriculum aids students in taking concrete actions toward their objectives (Mhlongo et al., 2025).

Participants indicated that Personal strategies to improve academic weaknesses, Support needed, Ways to overcome emotional weaknesses, improving social activities, and Ways to improve self-awareness are the methods of addressing learners’ weaknesses. As individual tactics, learners frequently mentioned time management, goal setting, seeking peer assistance, and intentional practice (reading, extra problems). These approaches are supported by research on student-centered techniques and individualized tutoring, which demonstrate quantifiable improvements when supports are customized to the learner’s needs and include feedback (Llerena Aguilar et al., 2023).

Throughout the interviews, learners requested personalized academic help (mentoring/tutoring); emotional/psychosocial support (emotion-regulation coaching, counseling); and more precise career/curriculum recommendations. The research (Llistosella et al., 2024) shows that the best ways to address complex, interrelated deficits are through multi-component school support, which combines social support, skill development, and structural classroom modifications.

Both human support (trusted adults, peer groups) and skill development (emotion management, mindfulness, cognitive reframing) were chosen by learners. Emotion-regulation therapies and mindfulness/self-esteem programs reduce anxiety and enhance classroom behavior, according to systematic reviews and recent RCTs/pilot trials. This suggests that schools might implement these as brief modules or incorporate them into life-orientation curriculum (Krobtrakulchai et al., 2024).

Students with participation anxiety benefited from low-stakes, scaffolded social opportunities (small clubs, peer mentoring, guided group work). Research on participation obstacles highlights both student skill development and contextual adaptations (sensory/space adjustments, teacher facilitation); combining the two strategies results in the biggest increases in meaningful engagement (Kara et al., 2025).

Goal-setting workshops, mentorship that emphasizes strengths, short classroom modules on growth mindset and self-regulation, and organized reflection exercises (such as diaries and guided self-assessment) are effective strategies that have been reported by both learners and literature. The best evidence for enhancing self-awareness and related results comes from multi-component school programs (resilience, self-esteem, and skill training) (Llistosella et al., 2024).

## 5 Limitations

The study’s findings cannot be generalized because it focused on six schools in Limpopo, leaving many schools out. Only 48 learners were involved out of thousands of them in Limpopo Province. Therefore, generalizability is difficult. Another limitation might be learners’ reluctance to disclose weaknesses due to fear of judgment, embarrassment, or stigma, and some learners may lack the ability to accurately identify their weaknesses, leading to inaccurate or incomplete data. Regarding the strategies to improve learners’ weaknesses, they might have lacked knowledge of effective strategies. Adolescents’ emotional and cognitive development is still ongoing. As a result, they might not fully grasp concepts such as “self-awareness,” and their job aspirations and self-perceptions might evolve over time. Responses in qualitative and participatory studies may be influenced by the researcher’s interpretation, the way questions are asked, or the facilitation. Despite efforts to maintain objectivity, theme development may reveal the researcher’s perspectives.

## 6 Conclusion

According to the study’s findings, learners have a developing but unequal grasp of self-awareness, which is crucial in determining their academic commitment and future goals. Self-reflection, self-belief, and knowledge of one’s own emotions and talents are fundamental to how learners perceive their experiences at school and beyond, according to findings on learners’ comprehension of self-awareness. This has achieved the purpose of exploring their self-awareness and themes related to their strengths and weaknesses. However, several weaknesses, such as emotional, social, academic, and contextual issues that prevent full engagement and outstanding performance, frequently undermine this understanding. Examining learners’ weaknesses reveals important emotional, social, physical, and academic issues that, if ignored, could prevent them from performing at their best. The challenges are, nevertheless, counterbalanced by the strengths recognized, which demonstrate the learners’ academic potential, resilience, and personal abilities.

Collectively, the themes and sub-themes offer a comprehensive understanding of learners’ personal and developmental journeys, highlighting the importance of fostering self-awareness while simultaneously enhancing students’ abilities and addressing their difficulties to support long-term personal growth and sustainable academic success. The participants, enrolled in Grades 9 through 11 and aged 14 to 17, are at a crucial stage of adolescence marked by identity construction, self-awareness, and future planning. As a result, their answers offer insightful insights into developmental processes related to individual strengths, shortcomings, and professional goals.

## Data Availability

The original contributions presented in the study are included in the article/supplementary material, further inquiries can be directed to the corresponding author.
